# The CD39-CD73-adenosine axis: a pivotal regulator of retinal homeostasis and diseases

**DOI:** 10.3389/fcell.2026.1893953

**Published:** 2026-09-08

**Authors:** Xinyi Chen, Jing Ji, Jiaxin Yao, Pengfan Chen, Jiali Zhang, Longqian Liu, Xiangyi Wen

**Affiliations:** 1 West China School of Medicine, Sichuan University, Chengdu, Sichuan, China; 2 Department of Ophthalmology, Department of Optometry and Visual Science, Laboratory of Optometry and Vision Sciences, West China Hospital, Sichuan University, Chengdu, Sichuan, China

**Keywords:** adenosine, CD39, CD73, purinergic signaling, retinal fundus diseases, therapeutic targeting

## Abstract

Extracellular purinergic signaling, mediated by nucleotides such as adenosine triphosphate (ATP) and adenosine, is a critical regulator of retinal homeostasis. Within the retina, this signaling pathway is indispensable for maintaining physiological functions. Dysregulation of extracellular purine balance is a key driver of retinal pathophysiology, contributing to inflammation, pathological angiogenesis, and neurodegeneration in diseases like diabetic retinopathy (DR), age-related macular degeneration (AMD), glaucoma, and retinopathy of prematurity (ROP). The hydrolysis of extracellular ATP to adenosine, primarily orchestrated by the sequential actions of NTPDase1/ENTPD1 (CD39) and ecto-5′-nucleotidase/NT5E (CD73), represents a fundamental mechanism for calibrating purinergic signals and shifting the microenvironment from pro-inflammatory to anti-inflammatory and protective. CD39 and CD73 are pivotal enzymes in regulating retinal purinergic equilibrium. Despite their well-characterized roles in tumor biology and cerebral pathophysiology, the expression, physiological functions, and pathological implications of CD39/CD73 in the retina remain understudied and lack systematic synthesis. This review synthesizes current understanding of the roles of CD39 and CD73 in retinal physiology and pathology, with a focus on their implications in DR, AMD, glaucoma, and ROP. Critically, this review provides the first systematic integration of their layer-specific spatial compartmentalization, context-dependent functional duality, and emerging metabolic roles in photoreceptor homeostasis. We also discuss emerging therapeutic strategies that target this pathway, addressing the growing need for translational research to preserve vision.

## 1 Introduction

Extracellular purinergic signaling, mediated by nucleotides like adenosine triphosphate (ATP) and adenosine, plays a critical role in retinal physiology and pathophysiology, regulating key processes including retinal blood flow (Losenkova et al., 2022; Lutty and McLeod, 2003), neuronal communication (Molcak et al., 2023), and immune responses (Molcak et al., 2023; Zeiner et al., 2019). Within the retina, this signaling pathway is essential for maintaining homeostasis (Fu et al., 2023). Alterations in extracellular purine balance, marked by excessive ATP accumulation or reduced adenosine bioavailability, are implicated in retinal pathophysiology, contributing to inflammation (Zeiner et al., 2019), neovascularization (Loukovaara et al., 2017), and apoptosis (Molcak et al., 2023). Notably, purine homeostasis is concurrently regulated by complementary mechanisms, including ATP hydrolysis (Kowal et al., 2015), nucleoside transporters (Lindberg et al., 2015), and receptor crosstalk (Molcak et al., 2023). These processes collectively control extracellular nucleotide and adenosine balance. Primarily, the regulation of extracellular purine levels is governed by enzymatic hydrolysis via the NTPDase1/ENTPD1 (CD39) and ecto-5′-nucleotidase/NT5E (CD73) (Ho et al., 2016), which operate sequentially: CD39 hydrolyzes ATP and adenosine diphosphate (ADP) to adenosine monophosphate (AMP), and CD73 subsequently converts AMP to adenosine (Losenkova et al., 2022; Rhett et al., 2014). Nucleoside transporters, such as equilibrative nucleoside transporter 1 (ENT1), complement this enzymatic axis by mediating the cellular uptake and clearance of extracellular adenosine, thereby terminating receptor signaling and regulating adenosine bioavailability (Liou et al., 2008; Bicket et al., 2016). Meanwhile, receptor-level crosstalk between adenosine-specific G protein-coupled receptors (P1 receptors) and nucleotide-activated purinergic receptors (P2 receptors) allows integration of ATP- and adenosine-derived signals, modulating downstream inflammatory, vasoactive, and neuroprotective outcomes in a context-dependent manner (Ye et al., 2021). Thus, CD39 and CD73 act as key regulators of purinergic signaling, preserving retinal homeostasis through the dynamic modulation of signal intensity and duration.

Although CD39 and CD73 are well-known for their roles in systemic immunity (Allard et al., 2017; Battastini et al., 2021), vascular function, and neuroprotection, their functions in the retina remain inadequately studied (Ye et al., 2021). Recently, growing evidence links these ectoenzymes and the adenosine receptor system to various fundus diseases, such as diabetic retinopathy (DR) (Elsherbiny et al., 2013; Ibrahim et al., 2011), retinopathy of prematurity (ROP) (Chen et al., 2017; Zhang et al., 2022), age-related macular degeneration (AMD) (Kaarniranta et al., 2023; Wang et al., 2023), and retinal inflammatory diseases (Wang et al., 2023; Sun et al., 2024; Vincent and Mohr, 2007). Nevertheless, a systematic synthesis of their roles in retinal physiology and pathology is currently lacking. Based on existing evidence, this review systematically outlines the contributions of CD39/CD73 to retinal processes and their involvement in ocular diseases, including current mechanistic insights, potential interventions, and outstanding questions related to it (Table 1).

**TABLE 1:** Distribution, functional roles, and disease associations of CD39 and CD73 in the retina.

Cell type	Distribution	Main physiological function	Related pathological role
CD39			
GCL/IPL	Synaptic terminals and cell membranes (McLeod et al., 2006)	Reduce excitotoxic ATP signaling (Shinozaki et al., 2023)	Glaucoma, contribute to ganglion cell stress (Shinozaki et al., 2023)
Optic nerve head	Vessels and nerve fibers (McLeod et al., 2006)	Regulate purinergic signaling, vascular function, and immune modulation (Shinozaki et al., 2023)	Glaucoma, may regulate the inflammatory response (Losenkova et al., 2022)
Retinal Vascular Endothelium	Luminal/abluminal surfaces of retinal vessels (Behdad et al., 2009)	Maintain anti-thrombotic and anti-inflammatory vascular homeostasis (Cao et al., 2020)	DR, promote thrombosis and inflammation (Zhou et al., 2020); ROP, neovascularization (Rivera et al., 2017)
Microglia	Cell surface and processes (Badimon et al., 2020); microglia-specific; Müller glia express ENTPD2 (Liang et al., 2023; Choi et al., 2023)	Form “purinergic junctions”, regulate microglial process ramification, suppress neuroinflammation (Abcouwer, 2017)	DR, neuronal degeneration, neuroinflammation (Bianco et al., 2022; Abcouwer, 2017); AMD, protect RGCs (Rodrigues-Neves et al., 2018)
Regulatory T Cells	Cell surface (Parodi et al., 2013)	Sustain local adenosine levels, curb excessive inflammation (Deaglio et al., 2007)	AMD, maintain immune homeostasis (Deaglio et al., 2007)
CD73			
RPE	Apical/basolateral membranes (Gagliardi et al., 2018)	Regulate ionic and metabolic equilibrium (Tang et al., 2024; Cheng et al., 2020)	AMD, impaired mitochondrial function (Brown et al., 2019)
Photoreceptors (Rods)	Highly expressed in cell bodies and processes (Zeiner et al., 2019; Gagliardi et al., 2018)	Protect against light-induced phototoxicity (Losenkova et al., 2022; Koso et al., 2009)	AMD and ROP, involved in photoreceptor degeneration and maturation (Koso et al., 2009; Brown et al., 2019)
OPL	Synaptic terminals and cell membranes (Losenkova et al., 2022)	Regulate photoreceptor-bipolar cell communication (Losenkova et al., 2022)	AMD, involved in neuroprotection (Losenkova et al., 2022)
INL	Cell surface	Support adenosine-mediated metabolic homeostasis	Regulate neurovascular homeostasis and inflammation (Losenkova et al., 2022; Di Virgilio and Adinolfi, 2017)
GCL/IPL	Apical/basolateral membranes of ganglion cells and IPL synapses (Losenkova et al., 2022)	Provide neuroprotection (Pietrucha-Dutczak et al., 2018)	Glaucoma, protect against excitotoxicity (Pietrucha-Dutczak et al., 2018)
Retinal Vascular Endothelium	Low	Maintain anti-thrombotic and anti-inflammatory vascular homeostasis	DR, reduce adenosine production (Li et al., 2025)
Microglia	Retinal parenchyma (Losenkova et al., 2022)	Form “purinergic junctions”, neuron-vascular communication (Losenkova et al., 2022)	Glaucoma, disrupted ATP/adenosine balance (Rodrigues-Neves et al., 2018); AMD, modulate microglial polarization (Meng et al., 2019)
Astrocyte Lineage	Peripheral Retina, transiently expressed on cell surface during development (Thompson et al., 2004)	May regulate developmental angiogenesis (Thompson et al., 2004)	ROP, neovascularization (Thompson et al., 2004)
Regulatory T Cells	Co-expressed with CD39 on cell surface (Parodi et al., 2013)	Mediate anti-inflammatory and immunomodulatory effects (Losenkova et al., 2022)	AMD, maintain immune homeostasis (Deaglio et al., 2007; Angioni et al., 2020)

Data were compiled from the following references: (Losenkova et al., 2022; Zeiner et al., 2019; Behdad et al., 2009; Liang et al., 2023; Choi et al., 2023; McLeod et al., 2006; Parodi et al., 2013; Koso et al., 2009; Gagliardi et al., 2018; Zhou et al., 2020; Badimon et al., 2020; Li et al., 2025; Deaglio et al., 2007; Bianco et al., 2022; Abcouwer, 2017; Di Virgilio and Adinolfi, 2017; Brown et al., 2019; Tang et al., 2024; Cheng et al., 2020; Angioni et al., 2020; Shinozaki et al., 2023; Pietrucha-Dutczak et al., 2018; Rodrigues-Neves et al., 2018; Meng et al., 2019; Rivera et al., 2017; Cao et al., 2020; Thompson et al., 2004).

CD39, NTPDase1/ENTPD1; CD73, ecto-5′-nucleotidase/NT5E; GCL, ganglion cell layer; IPL, inner plexiform layer; ATP, adenosine triphosphate; DR, diabetic retinopathy; ROP, retinopathy of prematurity; AMD, age-related macular degeneration; RGCs, retinal ganglion cells; RPE, retinal pigment epithelium; OPL, outer plexiform layer; INL, inner nuclear layer.

## 2 Expression and enzymatic properties of CD39 and CD73 in retina

### 2.1 CD39 and CD73 expression in the retina

CD39 and CD73 critically govern extracellular ATP and adenosine levels through spatiotemporally regulated hydrolysis. CD39 is predominantly localized to the luminal surface of retinal vascular endothelial cells comprising the inner blood-retina barrier (Fu et al., 2023; Behdad et al., 2009). Within the neural parenchyma, recent single-cell and spatial transcriptomic atlases have substantially refined the cellular resolution of ectonucleotidase expression: CD39 is preferentially enriched in microglia and vascular-associated cells, whereas Müller glia predominantly express NTPDase2, which hydrolyzes ATP to ADP without generating AMP (Liang et al., 2023; Choi et al., 2023; Iandiev et al., 2007; Ricatti et al., 2009). This restricted expression profile of CD39 extends to the optic nerve head (McLeod et al., 2006). In immune contexts, CD39 is expressed on retinal microglia and infiltrating macrophages, with higher levels in anti-inflammatory (M2) versus pro-inflammatory (M1) phenotypes (Allard et al., 2017). It also marks exhausted CD8^+^ T cells and regulatory T cells in chronic inflammation (Parodi et al., 2013).

CD73 exhibits a highly compartmentalized and layer-specific expression profile across the retina (Koso et al., 2009). It is polarically localized to the apical membrane of the retinal pigment epithelium (RPE) (Gagliardi et al., 2018). Notably, CD73 demonstrates peak enrichment within the photoreceptor layer (Zeiner et al., 2019), serving as a definitive surface marker for postmitotic photoreceptor precursors and mature rod cells (Gagliardi et al., 2018). Expression begins around birth and increases progressively to dominate over 90% of retinal cells in adults (Gagliardi et al., 2018), which is a conserved pattern from rodents to primates (Koso et al., 2009). Additionally, CD73 exhibits lower expression levels within the inner retinal layers.

The marked spatial segregation of CD39 and CD73 establishes functionally discrete purinergic microdomains. This compartmentalized arrangement implies that ATP hydrolysis and adenosine generation are differentially regulated across retinal layers: CD39 predominance in the inner retina favors rapid ATP clearance and limitation of purinergic inflammation, while CD73 enrichment in the outer retina facilitates local adenosine production (Losenkova et al., 2022; Liang et al., 2023; Choi et al., 2023). This layer-dependent functional dichotomy provides a mechanistic framework for understanding the topographic specificity of retinal diseases.

### 2.2 Enzymatic properties of CD39 and CD73

ATP ecto-nucleotidases are classified into three main types: CD39, NTPDase2 and CD73 (Losenkova et al., 2022; Fu et al., 2023). Among these, CD39 and CD73 form a critical enzymatic cascade for adenosine generation, which is critical for purinergic signaling (Zhou et al., 2020; Badimon et al., 2020). CD39, a transmembrane glycoprotein, catalyzes the hydrolysis of ATP and ADP to AMP with similar efficiency (Losenkova et al., 2022; Zeiner et al., 2019). CD73, attaching to the membrane (Heine et al., 2001), forms a homodimer essential for specific hydrolysis from AMP to adenosine (Sträter, 2006; Li et al., 2025; Matyash et al., 2017). Another member of NTPDase family, NTPDase2, have the high preference for the hydrolysis of ATP over ADP (Robson et al., 2006; Zimmermann et al., 2012). Co-localized with CD39 (Losenkova et al., 2022), this ectoenzyme presumably has functionality in the rapid scavenging of ATP in a neuronal environment, while the subsequent degradation of ATP derived ADP will occur with a considerable delay.

Specifically, CD39 initiates the cascade by hydrolyzing ATP in two steps (Losenkova et al., 2022; Antonioli et al., 2013):

ATP → ADP+Pi

ADP → AMP+Pi

This Mg^2+^/Ca^2+^-dependent process relies on conserved active-site residues to facilitate phosphate ester bond cleavage (Deaglio et al., 2007). By rapidly metabolizing ATP, this system prevents pathological ADP/ATP accumulation while generating adenosine in a tightly regulated manner. The efficiency of this system enables rapid responses to metabolic stress and establishes localized purine microdomains, crucial for retinal homeostasis (Molcak et al., 2023). Thus, the cascade converts a danger signal (extracellular ATP) into a protective mediator (adenosine), spatiotemporally fine-tuning purinergic signaling.

## 3 Function of CD39 and CD73 in physiological states of the retina

**FIGURE 1:**
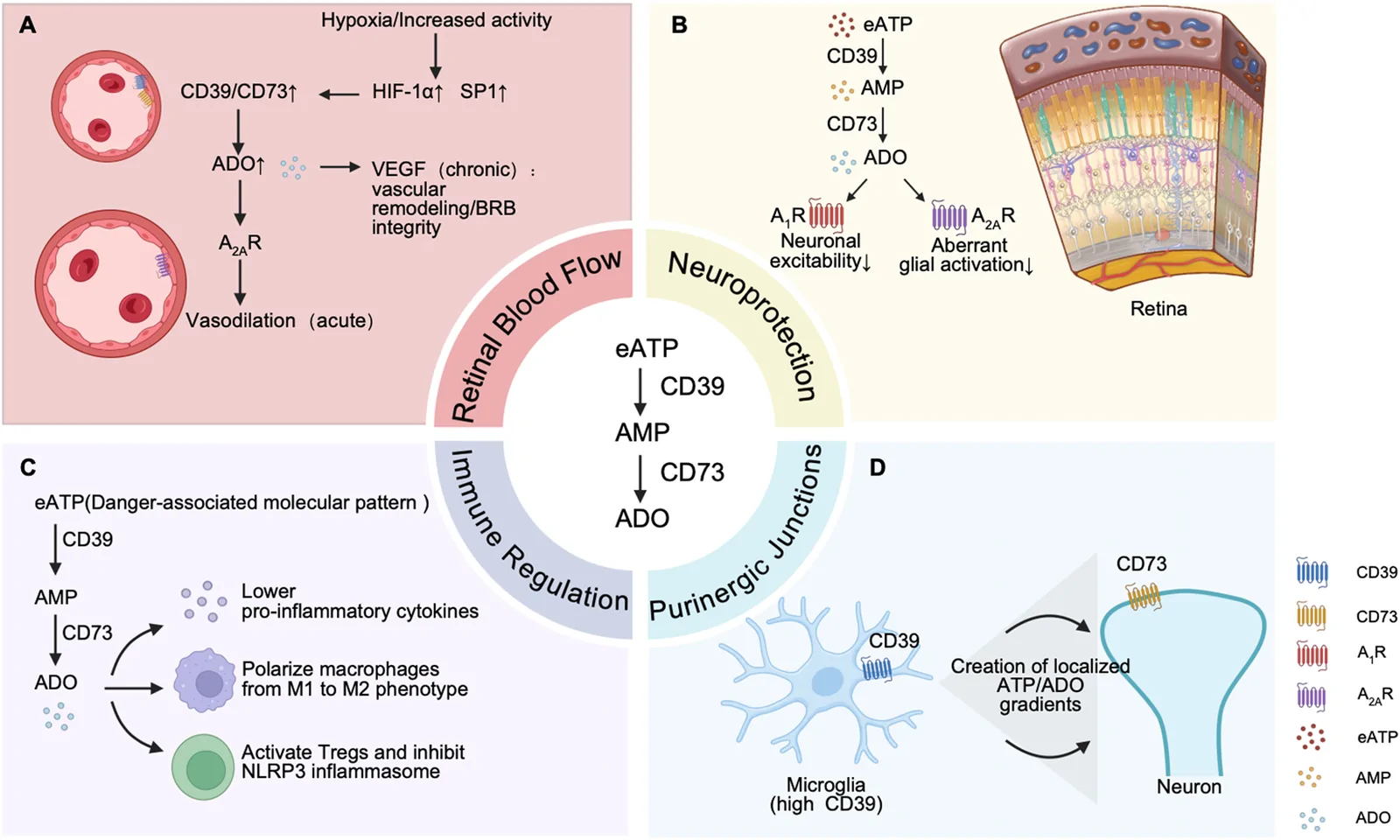
Physiological functions of the CD39-CD73-adenosine axis in the retina. **(A)** Retinal blood flow regulation: Hypoxia upregulates HIF-1α and SP1 to increase CD39/CD73 expression. Adenosine induces acute vasodilation and chronic VEGF-mediated vascular remodeling, maintaining BRB integrity. **(B)** Neuroprotection: The ATP-AMP-adenosine cascade modulates neuronal excitability via A_1_ receptor and suppresses aberrant glial activation via A_2A_ receptor, protecting photoreceptors and RGCs. **(C)** Immune regulation: CD39/CD73 convert danger-associated molecular pattern eATP to adenosine, reducing pro-inflammatory cytokines, polarizing macrophages to M2 phenotype, activating Tregs and inhibiting NLRP3 inflammasome. **(D)** Purinergic junction function: Microglia with high CD39 expression create localized ATP/adenosine gradients, regulating signaling and neuronal function. CD39, NTPDase1/ENTPD1; CD73, ecto-5′-nucleotidase/NT5E; HIF-1α, hypoxia-inducible factor-1α; SP1, specificity protein 1; VEGF, vascular endothelial growth factor; BRB, blood-retinal barrier; A_1_R, adenosine A_1_ receptor; A_2A_R, adenosine A_2A_ receptor; ATP, adenosine triphosphate; AMP, adenosine monophosphate; ADO, adenosine; Tregs, regulatory T cells; NLRP3, NOD-like receptor family pyrin domain-containing 3; RGCs, retinal ganglion cells. Created in BioRender. Chen, X. (2026) https://BioRender.com/cud45cj.

Under physiological conditions, CD39 and CD73 orchestrate retinal homeostasis by regulating the local balance between extracellular ATP and adenosine. This coordinated enzymatic cascade impacts multiple interdependent processes, ranging from vascular dynamics to neuronal and synaptic function, immune surveillance, and the formation of specialized purinergic microdomains. The following sections detail each of these mechanisms (Figure 1).

### 3.1 Regulation of retinal blood flow and metabolic homeostasis

The CD39/CD73 axis critically regulates retinal vascular homeostasis, ensuring perfusion matches the high metabolic demands of retinal neurons and photoreceptors (Avila et al., 2001). Upregulation of CD39/CD73 in high-demand regions rapidly generates adenosine during hypoxia or increased activity, facilitating immediate vasodilation to restore metabolic equilibrium (Eltzschig et al., 2004). Reduced metabolic demand correspondingly lowers adenosine production, maintaining vascular stability (Eltzschig et al., 2004).

Critically, CD39/CD73-mediated adenosine production constitutes an integral effector mechanism of retinal neurovascular coupling. Upon neuronal activation, Müller cells and neurons collaboratively release ATP into the perivascular space of retinal capillaries (Metea and Newman, 2006). Vascular endothelial cells and perivascular microglia expressing CD39 rapidly hydrolyze extracellular ATP and ADP to AMP; subsequent CD73 on endothelial cells and pericytes converts AMP to adenosine (Covarrubias et al., 2016). Notably, Müller cells themselves utilize NTPDase2 and nucleoside transporters to regulate extracellular purine levels, thereby contributing to the establishment of the layered retinal purine gradient (Robson et al., 2006). The generated adenosine then activates A_2A_ and A_2B_ receptors on pericytes and vascular smooth muscle cells, inducing vasodilation and matching local blood flow to elevated metabolic demand (Hein et al., 1999).

Beyond acute regulation, chronic adenosine signaling influences vascular remodeling by modulating angiogenic factors like vascular endothelial growth factor (VEGF), thereby affecting endothelial proliferation, migration, and blood-retinal barrier integrity (Loukovaara et al., 2017; Zhang S. et al., 2015). This integrates the CD39/CD73 axis in both immediate vascular modulation and angiogenic adaptation. Feedback mechanisms further refine the regulation: transient hypoxia stabilizes hypoxia-inducible factor-1α (HIF-1α), upregulating CD39/CD73 via specificity protein 1 (SP1) (Synnestvedt et al., 2002; Ai et al., 2019). Resultant adenosine amplifies A_2A_-mediated vasodilation, enhancing tissue oxygenation. This dynamic tuning synchronizes blood supply with metabolic flux, preventing ischemic injury and preserving neurovascular function. Together, the CD39/CD73–adenosine axis functions as a central signaling node embedded within the classical neurovascular coupling network, transducing neuronal metabolic activity into precisely calibrated vasodilatory responses.

### 3.2 Regulation of neuronal and synaptic activity

Retinal neurons, particularly photoreceptors and retinal ganglion cells (RGCs), are exceptionally sensitive to perturbations in the extracellular environment (Lu et al., 2015). Notably, the functional outcomes of extracellular ATP signaling are strictly context dependent, with divergent physiological and pathological effects dictated by its extracellular concentration and temporal duration of exposure.

Under physiological conditions, extracellular ATP is maintained at tightly regulated nanomolar concentrations. Released in a spatiotemporally constrained manner during synaptic transmission, glial-neuronal communication, and neurovascular signaling, it functions as a homeostatic signaling mediator rather than a cytotoxic effector (Losenkova et al., 2022). The CD39/CD73 axis provides essential neuroprotection by mitigating these events. The hydrolysis of CD39 reduces excitotoxic ATP availability and subsequently the hydrolysis of CD73 exerts neuroprotective effects via receptors A_1_ and A_2A_^52^. A_1_R activation inhibits presynaptic excitatory neurotransmitter release, dampening neuronal excitability (Gomes et al., 2011). Concurrently, A_2A_ receptor signaling suppresses aberrant glial activation, maintaining immune-neuronal homeostasis (Losenkova et al., 2022; Zhang et al., 2022).

Under pathological conditions characterized by sustained cellular stress, robust and prolonged ATP release exceeds the hydrolytic capacity of CD39, driving a pathological shift from homeostatic signaling to excitotoxic and pro-inflammatory damage (Molcak et al., 2023; Ye et al., 2021). Detailed downstream molecular mechanisms underlying this transition are elaborated in subsequent disease-specific sections.

CD39 on microglia and NTPDase2 on Müller glia together form a barrier that rapidly clears extracellular ATP, preventing its accumulation to neurotoxic levels. This confines purinergic signaling within a physiological window, preserving retinal homeostasis and blocking the transition from transient signaling to sustained excitotoxic injury.

### 3.3 Immune regulation and maintenance of anti-inflammatory balance

Under physiological conditions, extracellular ATP serves as a danger-associated molecular pattern (DAMP) that can bind to various P2 receptors on immune cells, triggering the release of pro-inflammatory cytokines and chemokines (Déchelle-Marquet et al., 2023). Given that excessive inflammation can lead to irreversible visual impairment in the retina, including the degeneration and apoptosis of irreplaceable photoreceptors and retinal pigment epithelial cells, and the disruption of the blood–retinal barrier (BRB) that allows inflammatory cell infiltration into the neuroretina (Mimura and Noma, 2025; Ozawa et al., 2011; Quinn et al., 2024), CD39 and CD73 play a critical role in immune regulation and maintenance of anti-inflammatory balance in retina. The rapid hydrolysis of ATP by CD39 is critical for containing inflammatory responses (Déchelle-Marquet et al., 2023; Fletcher, 2021). Adenosine generated by CD73 activates A_2A_ receptor on immune cells, thereby downregulating the production and release of cytokines such as interleukin-1β (IL-1β), tumor necrosis factor-α (TNF-α), and IL-6 (Ohta and Sitkovsky, 2001). Beyond modulating cytokine production, the adenosine also influences the behavior and phenotype of immune cells. Adenosine signaling can shift macrophage polarization from a classically activated, pro-inflammatory M1 state to an anti-inflammatory, repair-promoting M2 state. Furthermore, it modulates T cell function by limiting the activation of effector T cells while promoting regulatory T cell activity (Deaglio et al., 2007; Wang et al., 2013). Additionally, adenosine can inhibit the assembly and activation of inflammasomes, such as the NLR family pyrin domain containing 3 **(**NLRP3) complex (Ouyang et al., 2013), thereby reducing the release of additional pro-inflammatory mediators. These multifaceted immune-regulatory functions underscore the central role of the CD39/CD73 axis in maintaining retinal homeostasis, enabling regulation of immune responses in a manner that affords both rapid and sustained protection against inflammatory insults.

### 3.4 Purinergic junctions and regulation of ATP/adenosine gradients

An emerging concept in retina is the formation of purinergic junctions, which are microdomains where there is close apposition of cells with distinct expression profiles of CD39 and CD73 (Losenkova et al., 2022). These microdomains function as localized control centers for purinergic signaling, characterized by the physical proximity of CD39-rich cells to CD73-expressing cells (Deaglio et al., 2007). Such an arrangement facilitates substrate channeling, where AMP produced by CD39 activity is immediately available as a substrate for the subsequent reaction catalyzed by CD73 (Matsuoka and Ohkubo, 2004). The establishment of these local purinergic junctions is critical for creating steep gradients of extracellular ATP and adenosine (Losenkova et al., 2022). Within a typical purinergic signaling microdomain, CD39 rapidly hydrolyzes ATP to AMP, which diffuses proximally to adjacent cells expressing CD73 (Rahmaninejad et al., 2020; Mitchell and Reigada, 2008). Subsequently, CD73 catalyzes the conversion of AMP to adenosine, generating a focal adenosine surge (Rahmaninejad et al., 2020). Under normal physiological conditions, rapid hydrolysis of ATP minimizes the accumulation of this pro-inflammatory nucleotide, while the efficient generation of adenosine ensures that local adenosine receptor populations are continuously engaged (Ye et al., 2021). This precise tuning of purine levels is essential for modulation of neurotransmission, vascular tone, and regulation of inflammatory responses.

## 4 Function of CD39 and CD73 in pathological states of the retina

In pathological states, the homeostatic functions of the CD39-CD73-adenosine axis become dysregulated, contributing to disease progression across a spectrum of retinal disorders. Despite their distinct etiologies, DR, AMD, glaucoma, and ROP share several convergent purinergic disturbances: excessive extracellular ATP release from stressed or injured retinal cells, altered expression and spatial distribution of CD39 and CD73, disrupted ATP/adenosine equilibrium, and consequent amplification of inflammation, oxidative stress, vascular dysfunction, and neuronal injury. The following sections examine how these mechanisms manifest in each disease context, highlighting both common principles and disease-specific nuances.

### 4.1 Diabetic retinopathy

#### 4.1.1 Changes in the expression and distribution of CD39 and CD73

DR is one of the most common microvascular complications of diabetes, characterized by retinal vascular dysfunction, neuronal degeneration, and chronic inflammation (Altmann and Schmidt, 2018; Bianco et al., 2022). In the retinas of diabetic patients, significant changes occur in the expression and distribution of CD39 and CD73. Studies have shown that the density of CD39^+^/Iba-1^+^ resident microglia increases by 13% in type 1 diabetes (T1D) and 26% in type 2 diabetes (T2D) patients compared to non-diabetic controls, indicating activation in response to metabolic stress (Bianco et al., 2022; Hu et al., 2017). Concurrently, the intensity of quinolinic acid (QUIN) expression on CD39^+^ microglia is greatly increased, which may contribute to neuronal degeneration, as T1D and T2D retinas show significant losses of NeuN^+^ neurons (Bianco et al., 2022). The upregulation of CD39 on microglia might represent a compensatory mechanism to hydrolyze excessive extracellular ATP released from damaged cells, thereby attempting to limit neuroinflammation. However, the increased QUIN expression, a neurotoxic metabolite, on these CD39^+^ microglia indicates a potential pro-degenerative role, highlighting the complex and dual nature of CD39^+^ microglia in DR (Bianco et al., 2022; Abcouwer, 2017).

**FIGURE 2:**
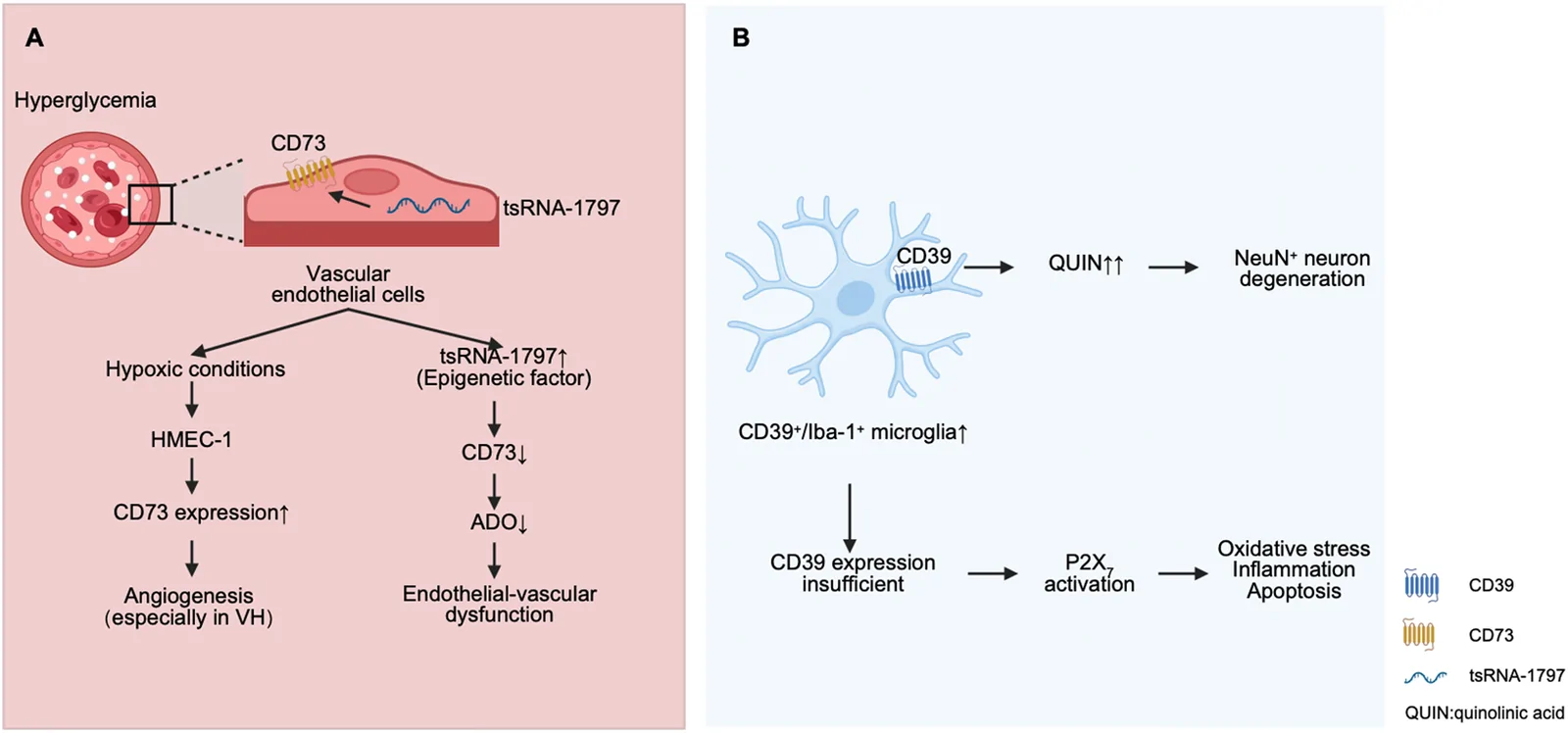
Related pathological role of CD39/CD73 in DR. **(A)** Under hyperglycemia, vascular endothelial cells show altered CD73 expression. Hypoxia upregulates CD73 in HMEC-1 to promote angiogenesis especially in VH. Elevated tsRNA-1797 epigenetically suppresses CD73, reducing adenosine production and leading to endothelial-vascular dysfunction. **(B)** Retinal microglia with high CD39 expression regulate neuronal survival through QUIN. Insufficient CD39 activates P2X_7_ receptor, inducing oxidative stress, inflammation and NeuN^+^ neuronal apoptosis. CD39, NTPDase1/ENTPD1; CD73, ecto-5′-nucleotidase/NT5E; DR, diabetic retinopathy; HMEC-1, human microvascular endothelial cells; VH, vitreous humor; QUIN, quinolinic acid; ADO, adenosine. Created in BioRender. Chen, X. (2026) https://BioRender.com/cud45cj.

Concurrently, CD73 expression is regulated by multiple pathological factors. In the pathological microenvironment of DR, hyperglycemia is the core factor initiating the cascade of retinal injury (Perrone et al., 2014). High glucose conditions regulate CD73 expression and activity through multiple mechanisms, thereby disrupting retinal purinergic metabolic balance (Li et al., 2025). Epigenetic factors, such as tsRNAs, were found as a novel epigenetic regulatory mechanism in DR by modulating CD73 expression (Li et al., 2025). Mechanistically, tsRNA-1797 (a transfer RNA-derived small RNA) disrupts purine metabolism by directly targeting CD73, reducing adenosine production. These regulatory mechanisms impact cellular behavior and contribute to retinal pathology. Under diabetic conditions, tsRNA-1797 expression is markedly upregulated in retinal tissue. Functional studies have demonstrated that tsRNA-1797 overexpression exacerbates dysfunction in retinal microvascular endothelial cells, while silencing tsRNA-1797 ameliorates endothelial dysfunction *in vitro* and inhibits retinal vascular dysfunction *in vivo* (Li et al., 2025) (Figure 2).

#### 4.1.2 Disruption of purine metabolism homeostasis and downstream pathological effects

As a highly active tissue of the central nervous system, the retina requires high oxygen consumption and metabolic demands. The retinal vascular systems transport nutrients to the retina and oxygen to the retina while facilitating the removal of metabolites. In retinal diseases, such as proliferative DR, this delicate balance is disrupted, leading to pathological outcomes including vision-threatening neovascularization (Fu et al., 2023).

Purinergic signaling is significantly altered in diabetes, with both P1 and P2 receptor sensitivity being affected, rather than just changes in expression (Zhou et al., 2020). Studies in animal models and human retinal cells have demonstrated that CD39 deficiency in the DR results in the accumulation of extracellular ATP, which subsequently activates the P2X_7_ receptor (Zhou et al., 2020; Déchelle-Marquet et al., 2023). The P2X_7_ receptor is a ligand-gated non-selective cation channel that mediates the effects of ATP. Activation of P2X_7_ receptors has been specifically implicated in diabetes-induced retinal microvascular dysfunction (Zhou et al., 2020), which induces multiple downstream events including oxidative stress, inflammatory responses, and cell death (Ho et al., 2016).

In the context of DR, CD73 upregulation has been linked to promoting non-VEGF-dependent neovascularization. Specifically, studies have shown that retinal microvascular endothelial cells (HMEC-1) under hypoxic conditions exhibit increased expression of CD73, adenosine, and VEGF (Ai et al., 2019). Simultaneously inhibiting SP1 and HIF-1α can effectively reduce the expression of CD39, CD73, adenosine, and VEGF, suggesting that CD73 contributes to angiogenesis through the CD39-CD73-adenosine pathway (Ai et al., 2019; Kishore et al., 2018). This pathological shift is exacerbated by a systemic imbalance within the purine-metabolizing enzyme network, especially in the vitreous microenvironment of DR patients with complications like vitreous hemorrhage (VH) (Zeiner et al., 2019). Studies have revealed elevated levels of soluble adenylate kinase, adenosine deaminase, CD73, and alkaline phosphatase in patients with DR accompanied by vitreous hemorrhage and elevated levels of ATP (Zeiner et al., 2019; Watanabe et al., 2005; García-Ramírez et al., 2008). Consequently, although CD73 activity is upregulated, the adenosine it produces may be rapidly catabolized by concurrently elevated adenosine deaminase, resulting in no net increase in adenosine bioavailability (Di Virgilio and Adinolfi, 2017). Instead, persistent ATP generation and accumulation shift purinergic signaling from an anti-inflammatory adenosine-dominated phenotype toward a pro-inflammatory state characterized by ATP regeneration. This proposed imbalance in the enzyme network explains the coexistence of elevated CD73 activity and increased ATP levels in the vitreous of DR patients, fostering an environment conducive to neovascularization.

### 4.2 Age-related macular degeneration

AMD is a progressive neurodegenerative disease characterized by the degeneration of RPE cells, photoreceptors, and choroidal vasculature in the macula, leading to central vision loss (Chen and Xu, 2015; Swaroop et al., 2007; Thomas et al., 2021). The pathogenesis of AMD involves complex interactions between genetic, environmental, and metabolic factors, with oxidative stress, chronic inflammation, and mitochondrial dysfunction playing central roles (Chen and Xu, 2015; Hsueh et al., 2022).

#### 4.2.1 Inflammation and immune disorders

Aberrant purinergic signaling significantly contributes to AMD through dysregulated activation of P2X_7_ receptor pathway (Calzaferri et al., 2020). As described in the context of DR, extracellular ATP accumulation can drive P2X_7_-mediated inflammation and cellular injury. In AMD, P2X_7_ activation assumes particular significance due to its coupling with the NLRP3 inflammasome in RPE cells and photoreceptors, triggering the release of IL-1β and IL-18 and amplifying local oxidative stress and tissue degeneration (Fletcher, 2021; Yang, 2017; Sekar et al., 2023). In a mouse model of dry AMD induced by sodium iodate (NaIO_3_), which causes RPE damage and subsequent retinal degeneration, P2X_7_ knockout mice were protected from retinopathy and showed reduced expression of inflammatory genes (NLRP3, IL-1β, IL-6) (Sekar et al., 2023). Thus, CD39 could serve as a negative regulator of P2X_7_-mediated pathology in AMD.

**FIGURE 3:**
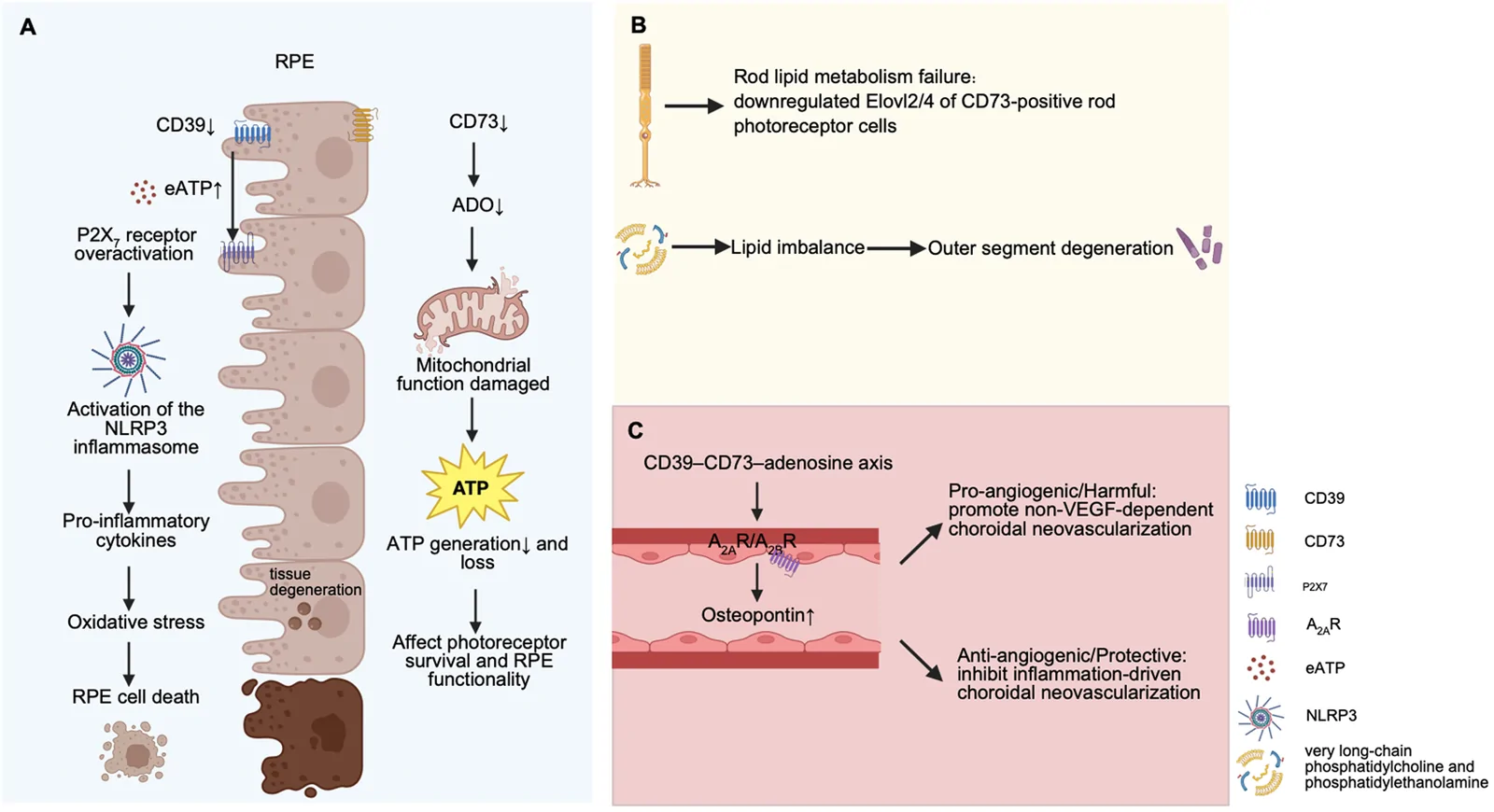
Related pathological role of CD39/CD73 in AMD. **(A)** In RPE cells, reduced CD39 causes P2X_7_ receptor overactivation and NLRP3 inflammasome assembly, releasing pro-inflammatory cytokines and inducing RPE cell death. CD73 deficiency impairs mitochondrial function, reducing ATP generation and exacerbating tissue degeneration. **(B)** CD73 downregulation disrupts rod lipid metabolism via reduced Elovl2/4 expression, causing outer segment degeneration. **(C)** The CD39-CD73-adenosine axis plays a dual role in choroidal neovascularization. CD39, NTPDase1/ENTPD1; CD73, ecto-5′-nucleotidase/NT5E; AMD, age-related macular degeneration; RPE, retinal pigment epithelium; NLRP3, NOD-like receptor family pyrin domain-containing 3; ATP, adenosine triphosphate; Elovl2/4, elongation of very long chain fatty acids protein 2/4; A_2A_/A_2B_, adenosine A_2A_/A_2B_ receptors; VEGF, vascular endothelial growth factor. Created in BioRender. Chen, X. (2026) https://BioRender.com/cud45cj.

Furthermore, CD39 and CD73 act synergistically in immune regulation. In dry AMD or photoreceptor degeneration, CD73 primarily mediates anti-inflammatory and immunomodulatory effects through immune cells such as Tregs (Antonioli et al., 2013; Deaglio et al., 2007; Xu et al., 2013). CD39 is also highly expressed on immune cells and acts in synergy with CD73 to sustain local adenosine levels and curb excessive inflammation (Allard et al., 2017; Antonioli et al., 2013; Deaglio et al., 2007). Thus, within the context of inflammation and immune regulation in AMD, CD39 and CD73 likely operate in a complementary manner to maintain immune homeostasis in the retinal microenvironment (Kerkelä et al., 2016) (Figure 3).

#### 4.2.2 RPE mitochondrial dysfunction and lipid metabolism disorder

In AMD, purinergic signaling dysregulation is implicated in mitochondrial dysfunction in both RPE and photoreceptors, contributing to the pathogenesis of the disease (Santos-Ferreira et al., 2015). Studies have demonstrated that CD73 deficiency reduces extracellular adenosine levels in the retina. This adenosine deficiency can impair the ability of RPE and photoreceptors to maintain mitochondrial membrane potential and ATP production (Manzar et al., 2020). Without sufficient adenosine, mitochondrial function declines, leading to increased oxidative stress and reduced energy production (Manzar et al., 2020). This impairment exacerbates the degenerative processes in AMD, affecting photoreceptor survival and RPE functionality (Brown et al., 2019).

CD73 may also participate in AMD pathology by influencing retinal cell lipid metabolism. The study employed cell sorting technology based on CD73 expression found that, during normal retinal development, the content of very long-chain phosphatidylcholine and phosphatidylethanolamine in CD73-positive rod photoreceptor cells significantly increased with development (Koso et al., 2009), consistent with elevated mRNA levels of Elovl2 and Elovl4 (Karan et al., 2005; Donato et al., 2018). In the NaIO_3_-induced photoreceptor degeneration model, specific changes in phosphatidylcholine and phosphatidylethanolamine levels within CD73-positive rod photoreceptor cells occurred 1 day before morphological damage appeared in the photoreceptor outer segments (Hamano et al., 2021). This suggests that lipid metabolism imbalance in CD73-positive photoreceptor cells under pathological conditions may precede structural damage and could be a significant cause of photoreceptor dysfunction and death. In AMD, abnormal lipid metabolism in RPE cells is a major cause of drusen formation, while photoreceptor cells have extremely high lipid metabolic demands (Tang et al., 2024; Cheng et al., 2020). Therefore, CD73 may participate in AMD pathology by regulating lipid metabolism in photoreceptor cells.

#### 4.2.3 Dual role of CD39 and CD73

In the context of choroidal neovascularization associated with wet AMD, the CD39/CD73-adenosine pathway may exert dual and opposing effects. Adenosine signaling through A_2A_ and A_2B_ receptors has been shown to promote angiogenesis (Antonioli et al., 2013; Weber et al., 2018; Montesinos et al., 2004). In valvular interstitial cells, stimulation of these receptors induces pronounced pro-degenerative responses, including upregulation of osteopontin (Weber et al., 2018), a multifunctional protein implicated in both angiogenesis and inflammatory processes. Therefore, upregulation of CD39 and CD73 within the choroidal vasculature or infiltrating immune cells in AMD may enhance adenosine generation, thereby potentiating neovascularization (Eltzschig et al., 2003). Conversely, adenosine also exhibits anti-inflammatory effects via A_2A_ receptor activation on immune cells, which may attenuate inflammation-driven neovascularization (Ohta and Sitkovsky, 2001; Yang et al., 2006; Zhou et al., 2005). This dual functionality complicates the therapeutic targeting of CD39 in wet AMD, as modulation of this pathway could either promote or suppress pathological neovascularization depending on the cellular milieu and specific patterns of adenosine receptor expression (Angioni et al., 2020).

### 4.3 Glaucoma

#### 4.3.1 RGC injury

**FIGURE 4:**
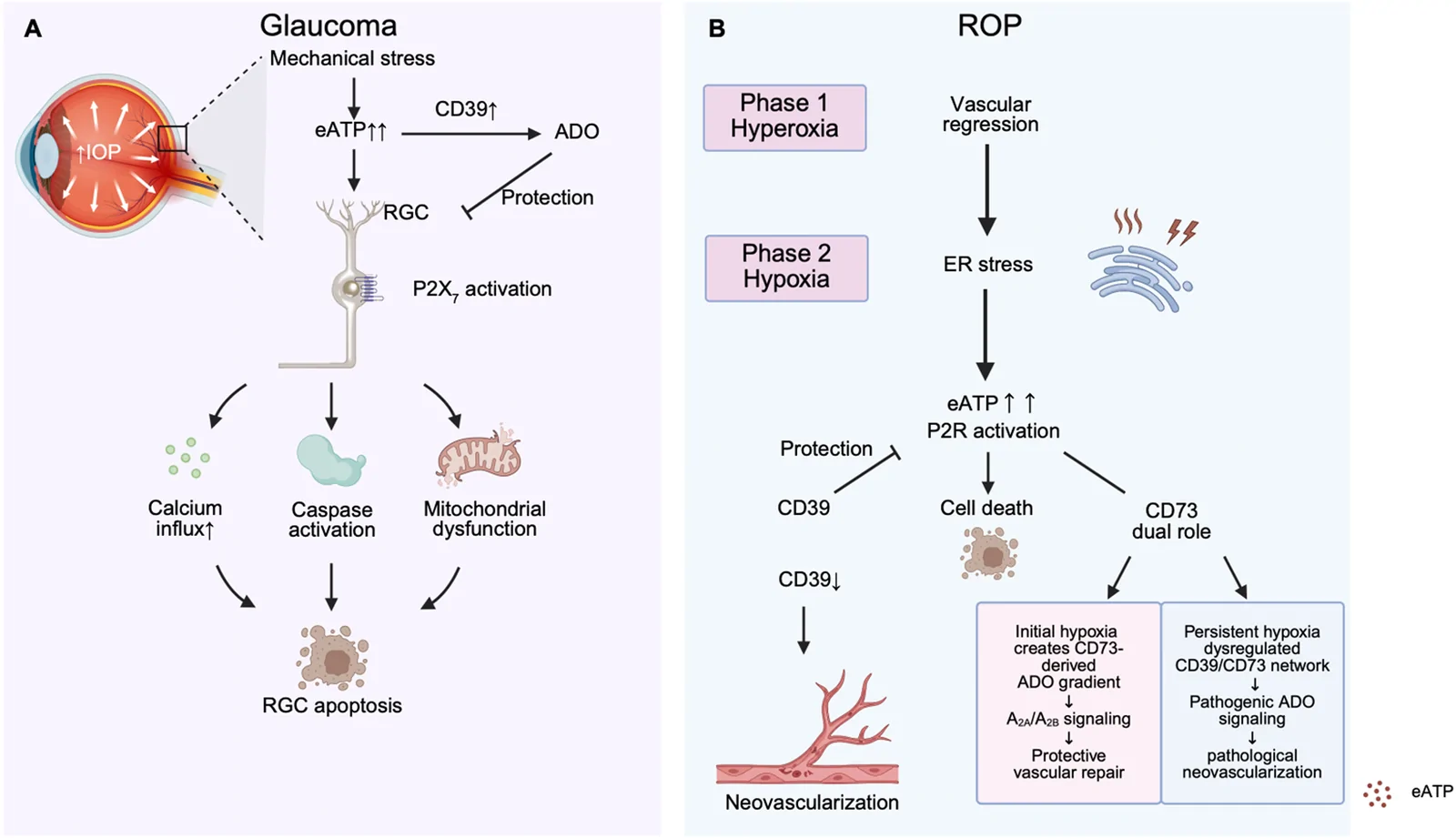
Related pathological role of CD39/CD73 in glaucoma and ROP. **(A)** In glaucoma, elevated IOP and mechanical stress increase eATP levels in RGCs. eATP activates P2X_7_ receptor, triggering calcium influx, caspase activation and mitochondrial dysfunction, ultimately leading to RGC apoptosis. CD39 upregulation partially converts ATP to adenosine for neuroprotection. **(B)** In ROP, hypoxia induces ER stress and elevates extracellular ATP. CD39 deficiency drives pathological neovascularization. CD73 plays a dual role: initial hypoxia promotes protective vascular repair, while persistent hypoxia leads to pathological neovascularization. CD39, NTPDase1/ENTPD1; CD73, ecto-5′-nucleotidase/NT5E; ROP, retinopathy of prematurity; IOP, intraocular pressure; eATP, extracellular adenosine triphosphate; ADO, adenosine; RGC, retinal ganglion cell; ER, endoplasmic reticulum. Created in BioRender. Chen, X. (2026) https://BioRender.com/cud45cj.

The ATP/CD39/CD73 axis demonstrates sensitivity to microenvironmental stress, undergoing significant modulation under pathological conditions such as elevated intraocular pressure (IOP) (Figure 4). Glaucoma, characterized by elevated IOP, progressive optic nerve degeneration, and RGC loss, involves pathological dysregulation of purinergic signaling (Shinozaki et al., 2023; Aslan et al., 2013; Pietrucha-Dutczak et al., 2018). Elevated IOP induces substantial ATP release from mechanically stressed retinal cells (such as Müller cells) (Shinozaki et al., 2023; Amato et al., 2023; Roberti et al., 2015), leading to accumulation of extracellular ATP. This excess ATP activates P2X_7_ receptors on RGCs, triggering pro-apoptotic cascades through pathological calcium influx, caspase activation, and mitochondrial dysfunction (Martucci and Nucci, 2019). Notably, retinal microglia contribute to this local purinergic regulation by extending and retracting their branched CD39^high^/CD73^low^ processes to form spatially arranged networks in the retinal parenchyma (Losenkova et al., 2022). These dynamic processes establish local “purinergic junctions” with CD39^low^/CD73^−^ neuronal cell bodies and CD39^high^/CD73^−^ retinal blood vessels, thereby facilitating neuron-vascular communication (Losenkova et al., 2022). Concurrently, CD39 expression is upregulated, which enzymatically degrades extracellular ATP to adenosine (Rodrigues-Neves et al., 2018). This catabolic activity counterbalances ATP-mediated excitotoxicity, thereby protecting RGCs from pressure-induced damage (Rodrigues-Neves et al., 2018).

Oxidative stress represents another critical mechanism underlying RGC injury in glaucoma. Elevated IOP, ischemia, hypoxia, and related factors enhance reactive oxidative species (ROS) production within retinal cells, overwhelming antioxidant defenses and inducing oxidative damage to lipids, proteins, and DNA, ultimately leading to RGC apoptosis (Hsueh et al., 2022; Tezel, 2006). Hence, by facilitating adenosine production, CD73 may contribute to antioxidative mechanisms in the glaucomatous retina, helping to protect RGCs from oxidative damage (Losenkova et al., 2022).

#### 4.3.2 CD39/

The chronic inflammatory microenvironment in the glaucomatous retina is a central mechanism driving progressive RGC loss, with aberrant microglial activation serving as a key instigator of neuroinflammation (Soto and Howell, 2014; Guo et al., 2022). Under physiological conditions, retinal microglia maintain a resting state, characterized by immune surveillance and neurotrophic support (Matyash et al., 2017). In response to glaucomatous insults such as elevated IOP or RGC injury, microglia become activated and adopt a pro-inflammatory M1 phenotype, releasing numerous inflammatory cytokines and ROS, which exacerbate RGC damage (Fernández-Albarral et al., 2024). Meanwhile, mechanistic studies demonstrate that IOP elevation alters microglial purinergic responses: murine microglia exposed to elevated hydrostatic pressure exhibit increased extracellular adenosine, upregulated CD39 expression, reduced equilibrative nucleotide transporter 2 (ENT2) density, and diminished adenosine deaminase (ADA) activity (Rodrigues-Neves et al., 2018; Huizinga et al., 2012). These changes synergistically drive adenosine accumulation: CD39 upregulation accelerates ATP hydrolysis, while ADA suppression reduces adenosine degradation and ENT2 downregulation limits cellular reuptake. CD73, through its generation of adenosine, may also play a pivotal role in modulating microglial polarization (Meng et al., 2019). Thus, CD39 and CD73 expression and activity can modulate local adenosine levels, influencing microglial function and phenotypic fate.

### 4.4 Retinopathy of prematurity

ROP is a vasoproliferative disorder of the developing retina, primarily affecting premature infants (Kumawat et al., 2021; Chen et al., 2024). It is characterized by an initial phase of retinal vascular obliteration due to hyperoxia, followed by a second phase of relative hypoxia that drives pathological neovascularization (Penn et al., 2008). The pathogenesis involves complex interactions between oxygen tension, VEGF, inflammation, and oxidative stress (Rivera et al., 2017; Tsang et al., 2019). The inflammatory mechanisms in ROP, particularly involving microglial activation and NLRP3 inflammasome signaling (Tsang et al., 2019; Rathi et al., 2017), share significant similarities with those previously described in AMD (see Section 4.2).

In the pathogenesis of ROP, the initial hyperoxic phase induces vaso-obliteration and retinal ischemia (Penn et al., 2008; Rivera et al., 2017), while subsequent hypoxia and associated oxidative stress can trigger endoplasmic reticulum (ER) stress in retinal cells. Both ischemia and ER stress promote the release of ATP from retinal cells, such as Müller glia (Zhang S. X. et al., 2015) (Figure 4). Accumulation of extracellular ATP activates P2 receptors, stimulating the release of inflammatory cytokines, endothelial proliferation and migration, and thereby driving pathological neovascularization (Zhang S. X. et al., 2015). For instance, in the oxygen-induced retinopathy (OIR) model of ROP, intravitreal injection of the glycolytic inhibitor 3-(3-Pyridinyl)-1-(4pyridinyl)-2-propen-1-one) (3PO) suppressed preretinal neovascularization by reducing ATP production in hypoxic cells (Hughes et al., 2025; Maitra, 2023).

CD39, by hydrolyzing ATP, counteracts these pro-angiogenic and pro-inflammatory signals. In the context of ER stress-induced ATP release, CD39-mediated hydrolysis may not only attenuate ATP-driven signaling but also mitigate ER stress itself by reducing P2 receptor-mediated calcium influx, thereby potentially promoting cell survival (Zhang S. X. et al., 2015; Cao et al., 2020). However, insufficient CD39 expression or activity may lead to inadequate clearance of excess ATP derived from both ischemic and ER stress pathways. While direct evidence of CD39 dysregulation in ROP is scarce, consistent with findings that CD39/CD73 were not among the hub genes identified in the OIR model (Xiong et al., 2022), reduced CD39^+^ microglial and regulatory cell activity is broadly linked to extracellular ATP accumulation, pro-inflammatory signaling, and neuronal damage across retinal and immune contexts. This failure can initiate a vicious cycle: ATP exacerbates ER stress, calcium overload, and inflammatory responses via P2 receptor signaling, resulting in further cellular damage and death (Rivera et al., 2017). These effects aggravate retinal ischemia, ultimately promoting more severe neovascularization. The function of CD73 in ROP may be dualistic: following the vaso-obliterative phase, retinal hypoxia upregulates CD73 expression (Synnestvedt et al., 2002). Initially, adenosine production via CD73 may serve a compensatory role, promoting vascular repair and regeneration through A_2A_/A_2B_ receptor signaling (Thompson et al., 2004). However, under sustained hypoxia, augmented CD73 activity, potentially coupled with altered expression of other purinergic enzymes may lead to dysregulated adenosine levels and prolonged receptor activation (Yan et al., 2019; Yang et al., 2015). This imbalance could divert adenosine’s action from reparative to pathogenic, driving aberrant neovascularization in the proliferative phase of ROP.

Furthermore, CD73 may influence ROP pathogenesis by modulating retinal neuronal development. Research suggests that CD73-positive rod photoreceptors display distinct lipid metabolic profiles during retinal development (Koso et al., 2009; Hamano et al., 2021). Preterm infants with ROP exhibit immature retinas and compromised photoreceptor development (Losenkova et al., 2022; Koso et al., 2009). As a marker of rod photoreceptors, dysregulated CD73 expression may disrupt lipid homeostasis, impairing photoreceptor maturation and thereby affecting neural signal transmission and integration, ultimately worsening visual outcomes in ROP (Losenkova et al., 2022).

## 5 CD39/CD73 as potential therapeutic targets

Based on the molecular mechanisms of CD39/CD73 and how its activation of key receptor systems impacts on key pathological processes, there might be a potential for employing regulation of this pathway for therapeutic benefit in retinal diseases.

### 5.1 Current therapeutic paradigms

The contemporary management of neovascular retinal diseases, such as proliferative DR and wet AMD, is dominated by therapies targeting VEGF (Arrigo et al., 2022; Xu et al., 2022). However, a substantial portion of patients exhibit a suboptimal response or develop tachyphylaxis over time. The therapeutic regimen, requiring frequent and burdensome intravitreal injections, poses a significant risk of complications (Xu et al., 2022). Beyond anti-VEGF monotherapy, treatments such as pan-retinal photocoagulation for DR and corticosteroids are also employed, which are associated with drawbacks including destructive effects on healthy retinal tissue and side effects like cataract formation and iatrogenic glaucoma (Arrigo et al., 2022). Critically, these established therapies primarily address the downstream consequences of disease (Muniyandi et al., 2023), namely, angiogenesis or edema, without directly targeting the upstream inflammatory and metabolic dysregulation that drives pathology. In non-neovascular conditions like glaucoma and dry AMD, therapeutic options are limited, focusing on slowing progression rather than reversing neurodegeneration or preventing inflammatory cell damage (Thomas et al., 2021).

### 5.2 CD39/CD73 as potential therapeutic targets

The CD39/CD73 ectoenzyme axis represents a compelling and complex therapeutic target across diverse retinal pathologies, with its modulation requiring precise disease-specific strategies due to the dichotomous roles of adenosine (Antonioli et al., 2013). In proliferative retinopathies, the hypoxic and inflammatory microenvironment drives overexpression of CD39 and CD73, culminating in excessive extracellular adenosine accumulation and neovascularization (Eltzschig et al., 2003). Preclinical studies utilizing potent inhibitors like PSB-12489 demonstrate reduced adenosine production and suppression of aberrant vessel growth in models of retinal stress (Sun et al., 2010; Stagg et al., 2011). Consequently, selective CD73 inhibition emerges as a primary therapeutic rationale, aiming to disrupt this pro-angiogenic cascade within the neovascular niche (Sun et al., 2010; Stagg et al., 2011).

Conversely, the therapeutic rationale fundamentally reverses for neurodegenerative conditions like glaucoma and dry AMD, where neuroprotection is paramount (Thomas et al., 2021; Bourne et al., 2016; Quigley and Broman, 2006). In glaucoma, enhancing the CD39/CD73 pathway to rapidly generate neuroprotective adenosine offers potential benefit, like promoting RGC survival (Lambiase et al., 2009). Agonists targeting A_2A_ and A_3_ receptors which stimulate adenosine action show promise in experimental models (Wilson and Di, 2012), improving RGC survival under hypoxic or high-pressure stress and reducing inflammation through distinct mechanisms. Their utility spans acute neuroprotection post-insult and chronic anti-inflammatory maintenance. However, significant knowledge gaps persist. Direct experimental evidence linking CD73-generated adenosine to RGC protection in glaucoma models is currently lacking. Similarly, in dry AMD, while chronic inflammation and complement activation drive drusen formation and RPE atrophy, and adenosine possesses clear anti-inflammatory potential, proteomic analyses of human AMD drusen have not identified significant alterations in CD39 or CD73 expression (Anderson et al., 2010; Bharti et al., 2022), highlighting a critical gap in understanding this axis’s specific role.

A major unresolved challenge in translating these preclinical concepts is the dualistic nature of adenosine. While adenosine exerts neuroprotective and anti-inflammatory effects via receptors under physiological or mildly stressed conditions, its sustained overproduction in chronic inflammatory microenvironments can promote pathological angiogenesis, fibrosis, and immunosuppression (Losenkova et al., 2022; Allard et al., 2017). This functional dichotomy means that simply enhancing or suppressing the CD39/CD73 pathway without precise spatiotemporal control could produce opposing outcomes depending on the disease stage and cellular context. Furthermore, the widespread expression of adenosine receptors across multiple retinal cell types raises concerns about off-target effects (Losenkova et al., 2022), while receptor redundancy, where multiple receptor subtypes mediate overlapping or opposing functions, complicates the prediction of therapeutic responses (Ye et al., 2023; Al-Attraqchi et al., 2019). Given the dualistic, context-dependent roles of adenosine in ROP, synthetic-biology circuits that integrate multiple pathological signals—for instance, ligand-triggered “AND gates” that engage downstream pathways only when several targets coincide—could, in principle, enable spatiotemporally controlled modulation of the CD39-CD73-adenosine axis. This concept, however, is largely unexplored in the retina, and its translational potential merits dedicated investigation.

The inherent complexity of purinergic signaling is further underscored by profound microenvironmental and cell type–dependent differences. Müller cells, microglia, and RPE cells exhibit distinct CD39/CD73 expression patterns dynamically regulated by hypoxia, oxidative stress, and cytokines (Chen et al., 2016; Faraoni et al., 2022). Consequently, achieving selective modulation without off-target effects necessitates sophisticated targeting strategies (Huang et al., 2019). Overcoming translational barriers requires addressing the poor pharmacological properties of existing modulators. Many small-molecule inhibitors, such as the CD39 inhibitor ARL67156, suffer from profound metabolic instability (*in vitro* half-lives <1 min), rendering them unsuitable for *in vivo* use (Rajakumar et al., 2010). Advanced drug delivery systems are therefore essential. Nanoparticle platforms and intravitreal sustained-release depots offer solutions by protecting therapeutic agents from degradation, extending half-life and bioavailability, and enabling months-long therapeutic levels, reducing injection frequency (Adamiak et al., 2019; de Leve et al., 2019).

Combination therapeutic approaches hold significant promise. Synergy exists between CD73 inhibition and anti-VEGF therapies in neovascular diseases like wet AMD and proliferative DR (Bradley et al., 2007; Liu et al., 2024; Fu et al., 2024). Preclinical models show combined treatment yields greater neovascular suppression than monotherapy, potentially countering VEGF escape mechanisms by attenuating the inflammatory drive undermining anti-VEGF efficacy and stabilizing the vascular microenvironment (ElSheikh et al., 2022).

Emerging gene and cell-based strategies offer novel avenues for long-term modulation. CRISPR/Cas9 gene editing enables precise targeting to either suppress CD73 in neovascular contexts or enhance protective pathway components in neurodegeneration (Jinek et al., 2012). Stem cell-based approaches, particularly mesenchymal stem cells genetically modified to overexpress factors like pigment epithelial-derived factor (Jones et al., 2017), demonstrate potential in DR models, reducing VEGF and neovascularization while promoting neuroprotection. However, it is critical to acknowledge that these approaches remain largely preclinical, and their translation is hindered by substantial barriers, including concerns over tumorigenicity, immune rejection, poor cell survival and integration post-transplantation, and the lack of standardized manufacturing protocols (Mount et al., 2015; Neofytou et al., 2015; Su et al., 2025).

## 6 Future directions

Despite compelling preclinical efficacy of the CD39/CD73 purinergic axis in rodents, translational hurdles persist. Extrapolating rodent pathophysiology to humans necessitates advanced human-relevant platforms and validation in non-human primates, which remain scarce (Kurago et al., 2023). Induced pluripotent stem cell (iPSC)-derived retinal organoids offer a promising human model for evaluating CD39/CD73 modulation, enabling CRISPR-Cas9-mediated mechanistic studies and compound screening (Allard et al., 2017). However, a key unresolved question is whether CD39/CD73 inhibition or selective A_2A_R blockade will confer clinical benefit alone or synergize with established therapies in treatment-refractory patients.

Therapeutic advancement requires progress in two areas: First, developing validated non-invasive biomarkers is critical but lacks clinical evidence correlating them with disease severity or therapeutic response in human retinopathies (Li et al., 2018; Kulkarni et al., 2025). Second, innovative delivery strategies capable of efficiently traversing or bypassing the BRB must be developed (Ben-Arzi et al., 2022). Nanocarriers with retinal tropism or engineered stem cells for localized adenosine modulation warrant exploration to achieve targeted efficacy while minimizing systemic effects (Ben-Arzi et al., 2022).

Collectively, the available evidence establishes the CD39-CD73-adenosine axis as a fundamental regulator of retinal homeostasis. This central role stems from its unique capacity to dynamically govern the balance between extracellular ATP and adenosine. Beyond sequential ATP hydrolysis, the spatially restricted distribution and hypoxia/cytokine-regulated expression of CD39 and CD73 enable precise and context-dependent control of neuroprotection, vascular tone, and immune homeostasis within the retinal microenvironment. Based on its functions, targeting the CD39/CD73 pathway presents as an attractive therapeutic avenue to be further explored across diverse retinal pathologies.
